# To prone or not to prone ARDS patients on ECMO

**DOI:** 10.1186/s13054-021-03675-6

**Published:** 2021-08-31

**Authors:** Oriol Roca, Andrés Pacheco, Marina García-de-Acilu

**Affiliations:** 1 Servei de Medicina Intensiva, Hospital Universitari Vall d’Hebron, Barcelona, Spain; 2 Centro de Investigación Biomédica en Red de Enfermedades Respiratorias (CibeRes), Madrid, Spain; 3 Departament de Medicina, Universitat Autònma de Barcelona, Bellatera, Spain

## Abstract

This article is one of ten reviews selected from the Annual Update in Intensive Care and Emergency Medicine 2021. Other selected articles can be found online at https://www.biomedcentral.com/collections/annualupdate2021. Further information about the Annual Update in Intensive Care and Emergency Medicine is available from https://link.springer.com/bookseries/8901.

## Introduction

The prone position is recommended as a supportive therapy in patients with moderate- to-severe acute respiratory distress syndrome (ARDS). It is usually associated with improved oxygenation and pulmonary mechanics as the result of a more homogeneous distribution of mechanical forces and better ventilation/perfusion (V/Q) matching. These effects lead to a lower risk of aggravating preexisting lung injury and, ultimately, a decrease in mortality. Despite widespread use of the prone position in patients with ARDS, even in awake non-intubated spontaneously breathing patients, its use dramatically decreases once the patient has been placed on extracorporeal membrane oxygenation (ECMO). In this chapter, we discuss the available evidence regarding use of the prone position in ARDS patients treated with ECMO.

## Physiological effects of prone position in patients with ARDS

The physiological effects of the prone position have been well described [1].

However, individual responses to the prone position may vary from one patient to another or even in the same patient at two different time points of his/her course in the ICU.

### Effects on respiratory mechanics and ventilation–perfusion ratio

Normally, the prone position decreases chest wall compliance [2] as a result of the limitation of abdominal expansion from contact with the bed and the fact that the posterior chest wall is less compliant. By contrast, the prone position generates a more homogeneous distribution of stress and strain in the lung parenchyma [3]; therefore, it may lead to more homogeneous inflation of the lung, decreasing the risk of tidal hyperinflation of non-dependent lung regions while simultaneously decreasing the cyclic opening and closing of alveolar units of the dependent lung. Hence, the prone position generates opposite effects on the chest wall and lung compliance. It should also be noted that the prone position may increase lung recruitment, defined as the total number of opened alveolar units. This effect is because the dorsal mass of the lung is greater than the ventral and not because there is any change in the average density of the lung, which remains unchanged regardless of the patient’s position. Finally, we should also remember that these changes in regional ventilation associated with prone position lead to a more homogeneous V/Q distribution [4] as perfusion remains mainly in the dorsal regions of the lungs when the patient is prone.

### Effects on gas exchange

The prone position may improve oxygenation as a result of the mechanisms mentioned earlier (more alveolar units open, better V/Q matching, and lower chest wall compliance of the anterior wall). However, the prone position may also have effects on the partial pressure of carbon dioxide in the arterial blood (PaCO2) levels. Indeed, patients who responded to decreased PaCO2 while maintaining the same minute ventilation presented better outcomes [5]. These changes have been associated with increased lung recruitment [6].

### Hemodynamic effects of prone position

Prone position has also been associated with right ventricular unloading, which leads to an increase in the cardiac index and a decrease in heart rate [7]. This is easily explained if we consider that hypoxemia, hypercapnia, and high driving and plateau pressures have been described as risk factors for acute cor pulmonale in ARDS patients [8] and could be reduced by the use of the prone position. Importantly, it may also partially explain the survival improvement described with the prone position in patients with ARDS [9], as no association between oxygenation improvement and survival has been observed [10].

### Effects on hospital-acquired respiratory infections

Another potentially significant effect of prone positioning is that, for anatomical reasons and the effect of gravity, when the patient is in the prone position, the dorsal part of the lung remains higher than the mouth, which favors the drainage of respiratory secretions. However, in an ancillary study of the PROSEVA (Proning Severe ARDS Patients) trial, prone positioning was not associated with a reduced incidence of ventilator-associated pneumonia (VAP) [11].

## Indications and contraindications

### Indications

According to the inclusion criteria used in the PROSEVA study, one may accept that the prone position is indicated in ARDS patients with a ratio of arterial oxygenation to fraction of inspired oxygen (PaO2/FiO2) <150 mmHg [9]. However, despite the observed mortality benefits, the results of a large multicenter observational study published 5 years after the PROSEVA trial to determine the prevalence of use of the prone position in ARDS, showed that the prone position was only used in 33% of patients with severe ARDS [12]. Thus, there is a critical gap between the evidence of mortality improvement and actual use of this management strategy. This gap is mainly due to the increase in workload, the absence of trained staff to perform the maneuver, and the possibility that it is still considered as a rescue maneuver that should be applied only to patients who present with refractory hypoxemia. However, the prone position may decrease mortality in patients with mild-to-moderate ARDS [13]. During the coronavirus pandemic, the results of some studies showed that the prone position was more widely used regardless of the severity of ARDS [14] and it was also used in non-intubated patients [15]. In fact, the prone position has been shown to decrease inspiratory effort and lung stress and to improve gas exchange while attenuating systemic inflammation in patients with ARDS [16]; the same effects might apply in awake patients.

### Contraindications

The absolute contraindication to using the prone position is the presence of unstable spinal fractures. All other contraindications are relative; therefore, decisions to use the prone position should be individualized. These relative contraindications include hemodynamic instability, unstable large bone or pelvic fracture, open abdominal wounds, increased intracranial pressure, or a risk of intracranial hypertension without adequate intracranial pressure monitoring. Although extra corporeal membrane oxygenation (ECMO) has not traditionally been considered a contraindication for prone positioning, proning is only used in 15% of patients who need to be placed on ECMO [17]. Several reasons may explain why the prone position is not continued when ECMO is started. First, there is a risk of ECMO-related complications when the patient is in the prone position. Second, is the fact that these patients were categorized as non-responders in the prone position in terms of oxygenation (this is the main reason for ECMO initiation). However, it is worth noting that the prone position has several essential benefits beyond oxygenation improvement, which may explain the survival benefit observed in prone patients; therefore, the absence of an oxygenation improvement after proning may not be sufficient to decide to discontinue the technique. Third, the fact that the patients were not proned before ECMO may partially explain why it is not used in these patients.

## How to perform prone positioning in ECMO patients

The prone position maneuver in patients treated with ECMO should not differ from that performed on non-ECMO patients. However, more staff members should participate in the maneuver [18]. Between four and eight persons will be needed depending on the experience of the team and the body mass index of the patient.

One person should be dedicated to managing the head of the patient and the artificial airway. In the case of an ECMO jugular cannula, this person will also control this cannula during the procedure. This person coordinates the entire prone position procedure. Another person must assess the correct functioning of ECMO and take care of the femoral ECMO lines. Finally, between one and three staff members on each side of the bed should perform the turning. During the proning maneuver, special attention needs to be paid to the ECMO flow and the integrity and potential displacement of the ECMO lines. Indeed, as the turning could be done with two persons on each side of the bed, another person could be in charge of fixing the cannulas at the insertion site in case of femoral insertion (jugular cannula will be controlled by the person allocated to the head of the patient).

Another critical issue is the direction of the turning. It has been proposed that turning should prioritize the reinjection line of VV-ECMO or the central venous lines, leaving them on the top during the turning, especially in patients with femorojugular access. It is essential to check the appropriate length of all the lines (ECMO, central venous, arterial, and ventilator circuits) before starting the maneuver. It should also be noted that the use of pillows is necessary to avoid compression of the femoral cannulas and to facilitate correct assessment of the insertion site to detect any bleeding.

### Clinical evidence of benefit from the prone position in patients treated with ECMO

Evidence regarding use of the prone position in patients treated with ECMO is continuously growing. Several studies have reported improvements in oxygenation [18–24] and respiratory system compliance (Crs) [18, 24, 25] after proning (Table 1). Improvement in respiratory mechanics, when it was specifically defined as an increase in Crs >3 ml/cmH2O (which represents a tidal volume increase of approximately 40 ml), was associated with a higher body mass index, more frequent viral pneumonia, shorter ECMO duration, and lower dorsal tidal volume distribution [25]. Interestingly, this higher increase in Crs observed in mechanical responders persisted for up to 6 h after returning to the supine position. These patients also presented a concomitant decrease in PaCO2 with no changes in the ventilator settings of sweep gas flow [25].

**Table 1:** Summary of key studies on prone positioning in patients with acute respiratory distress syndrome (ARDS) treated with extracorporeal membrane oxygenation (ECMO)

Reference	Type of study	Patients included	Prone position characteristics	Main results	Adverse events
Kipping et al. [19]	Retrospective cohort	12	Duration of prone sessions: 8 h Number of prone sessions: median of 6 (IQR 4–8) Total number of prone sessions: 74	58% reported an improvement in the PaO2/FiO2 ratio > 20% No change in MAP, HR, but mPAP significantly increased during proning and decreased after proning Dose of norepinephrine could also be decreased	One lost NGT Bleeding from ECMO cannulation sites (11/74) or tracheal tube (10/74) or central venous lines (8/74) or chest tubes (10/74) One endotracheal tube obstruction One pulmonary embolism Drop in SpO2 > 2% (10/74) One temporary reduced blood flow of ECMO Hemodynamic instability (7/74) Temporary bradycardia (3/74)
Masuda et al. [20]	Cohort	5	Duration of prone sessions: mean 15.3 ± 0.5 h Number of prone sessions: mean 1.8 ± 0.8	Oxygenation improvement: PaO2/FiO2 ratio in supine 143 ± 38 mmHg vs. prone 263 ± 99 mmHg	None
Guervilly et al. [21]	Prospective cohort	15	Initiation after a median of 9 (IQR 5–10) days on ECMO Duration of prone sessions: 12 h Number of prone sessions: 1.4 per patient Total number of prone sessions: 21	Oxygenation improvement: PaO2/FiO2 ratio increased from 103 (78–135) vs. 160 (96–215); *P* = 0.007 The oxygenation improvement persisted after returning the patients to the supine position No changes in PaCO2 and Crs were observed	No major adverse events Variations in ECMO flow were small (1.6 ± 4% compared to baseline) Two patients required crystalloid infusions of 500 ml for MAP < 65 mmHg during proning One pneumothorax occurring during proning was diagnosed and drained only after returning to supine position
Kimmoun et al. [18]	Retrospective cohort	17	Initiation after a median of 6 (4–12) days on ECMO Duration of prone sessions: 24 h Total number of prone sessions: 27	Oxygenation improvement: PaO2/FiO2 ratio in supine 111 (IQR 84–128) mmHg vs. at the end of prone session 173 (120–203) mmHg Oxygenation improvement occurred more frequently in patients who were proned after 7 days of ECMO therapy Improvement in Crs: From 18 (12–36) to 32 (15–36) ml/ cmH2O 24 h after the return to supine position, tidal volume was increased from 3.0 (2.2–4.0) to 3.7(2.8–5.0) ml/kg No correlation was observed between the oxygenation improvement and the amount of non-aerated lung tissue in the CT scan	One membrane thrombosis, one drop in ECMO blood flow
Lucchini et al. [22]	Retrospective cohort	14	Duration of prone sessions: median 8 h (IQR 6–10) Number of prone sessions: (median 1—IQR 1–1.5) Total number of prone sessions: 45	Oxygenation improvement: PaO2/FiO2 ratio in supine 123 (IQR 82–135) mmHg and at the end of prone session 149 (90–186) mmHg This improvement was not maintained when the patient was turned to supine 113 (74–182) No significant hemodynamic variations (HR, SAP, PAPm, CO, PWP, SvO2)	None
Rilinger et al. [26]	Retrospective cohort propensity score matched	38	Initiation after a median of 1.7 (0.5–5.0) days on ECMO Duration of prone sessions: median 19.5 (IQR 16.8–20.8) hours Number of prone sessions: 2 (1–3)	No difference in hospital survival (36.8% vs. 36.8%, *P* = 1.0) No difference in ECMO weaning rate (47.4% vs. 44.7%, *P* = 0.82) Hospital survival was superior in the subgroup of patients treated with early proning (< 17 h) as compared to late or no proning (81.8% vs. 33.3%, *P* = 0.02) 60-day mortality was 18% for the early proning and 65% for the late and no proning group, respectively (*P* = 0.027) Survival rate of early proning was higher compared to late proning or no prone (81.8% vs. 18.5% and 36.7%, *P* < 0.001 and *P* = 0.003, respectively)	No relevant complications
Franchineau et al. [25]	Prospective cohort	21	Duration of prone sessions: 16 h	Static Crs during proning increased from 23 (17–29) to 27 (20–37) ml/cmH2O (*P* < 0.01) 13 (62%) patients increased their static Crs by 3 ml/cmH2O after proning on ECMO (mechanical responders) EELI was redistributed from ventral to dorsal regions during proning Optimal PEEP determined by EIT was lower in prone position (14 (12–16) vs. 10 (8–14) cmH2O	None
Garcia et al. [23]	Retrospective cohort	14 (SARS- CoV-2)	Duration of prone sessions: median 16 h (IQR 15–17) Total number of prone sessions: 24	Oxygenation improvement: PaO2/FiO2 ratio in supine 84 (IQR 73–108) vs 112 (83–157) after proning The median PaO2/FiO2 ratio improvement after proning was 28% [2–36]. 62.5% high responders (increase PaO2/ FiO2 ratio > 20%), 16.7% moderate- responders (increase PaO2/FiO2 < 20%), and 20.8% non- responders (decrease PaO2/FiO2) Patients in the prone ECMO group were less likely to be weaned from ECMO, and 28-day mortality rate was significantly higher	Three minor hemorrhages at site of cannula insertion Three moderate flow drops of VV-ECMO that required fluid resuscitation
Giani et al. [24]	Multicenter retrospective cohort propensity score matched	240 patients (66 matched pairs)	Initiation after a median of 4 (IQR 2–7) days on ECMO Duration of proning: mean 15 (12–18) h Total number of prone sessions: 326	Improvement in oxygenation, intrapulmonary shunt fraction and static Crs that persisted after supination Minor differences in hemodynamics (mPAPm and PWP were slightly higher during proning and HR was lower) Lower hospital mortality in proned patients (OR = 0.50, 95%CI: 0.29–0.87) PS matched cohort: proned patients had a lower mortality (30% vs. 53%, *P* = 0.0241) proned patients had a longer duration of ECMO (16 vs10 days, *P* = 0.0344)	No major complication 6% minor complications Six procedures aborted due to respiratory or hemodynamic instability during prone positioning

*IQR* interquartile range, *PaO2/FiO2* ratio of arterial oxygenation to fraction of inspired oxygen, *MAP* mean arterial pressure, *HR* heart rate, *mPAP* mean pulmonary arterial pressure, *NGT* nasogastric tube, *PaCO2* partial pressure of carbon dioxide in the arterial blood, *Crs* respiratory system compliance, *SAP* systolic arterial pressure, *CO* cardiac output, *EELI* end-expiratory lung impedance, *PEEP* positive end-expiratory pressure, *EIT* electrical impedance tomography

Other important conclusions can be drawn from studies that used electrical impedance tomography (EIT) to monitor ECMO patients during proning. First, the optimal positive end-expiratory pressure (PEEP) levels in the prone position, determined by EIT and defined as the minimum sum of collapse and overdistension in a decremental PEEP maneuver, were significantly lower than in the supine position [25]. Moreover, as the prone position increases lung homogeneity, the same PEEP levels are less likely to generate tidal hyperinflation. Finally, it could also be observed that the lower levels of PEEP needed during proning and the associated changes in regional ventilation distribution were independent of the mechanical response. Thus, mechanical changes after proning are not good surrogates for proning- induced ventilation distribution changes. Indeed, changes in regional ventilation were also observed, even in patients who presented with lower Crs after proning.

Two studies have used the prone position as rescue therapy [18, 21]. The first study included patients who had failed to wean from ECMO after 7 days or those who had a PaO2/FiO2 <85 mmHg despite an FiO2 of 1 on both ventilator and ECMO, combined or not with plateau pressure >25 cmH2O [18]. The second study included patients who met at least one of the following three conditions: PaO2/FiO2 <70 mmHg despite maximal oxygenation, plateau pressure >32 cmH2O, or failure to wean from ECMO after 10 days of support [21]. In both studies, improvements in oxygenation were observed. It should be noted that, in the study by Kimmoun et al. [18], prolonged prone position sessions of 24 h were used, and the results showed improvement in both oxygenation and respiratory mechanics at the end of the prone session. Similarly, the results of a more recent study showed that improvements associated with the prone position continued to evolve during the 16-h sessions in the prone position, emphasizing the need for longer durations of prone sessions to achieve the maximal benefit [25].

Three studies have analyzed the effect of proning ARDS patients receiving ECMO [23, 24, 26]. The first was a single center retrospective study that compared 14 patients with ARDS on ECMO who were proned with 11 who were not proned [23]. Patients who were proned were less likely to be weaned from ECMO and had a higher 28-day mortality rate. However, there was an important selection bias as the prone position was initiated if the PaO2/FiO2 ratio was <80 mmHg despite an FiO2 of 1 both on the ventilator and the ECMO circuit and in the case of consolidation of more than 50% of the lung volume. The second study analyzed 38 matched pairs of patients with ARDS [26]: no differences in ECMO weaning rates or hospital survival were observed. However, by contrast to the results of the study by Kimmoun et al. [18], which reported that oxygenation improvements (increase >20% in the PaO2/FiO2 ratio) were more frequently observed in patients who had been treated for 7 days or more with ECMO therapy, patients who were proned within the first 17 h of ECMO therapy had lower in-hospital and 60-day mortality rates compared to those who were proned later or those who were not proned at all [26]. Finally, in a multicenter retrospective study of 240 patients with ARDS receiving ECMO [24], multivariate analysis showed that the prone position was associated with lower hospital mortality. Moreover, in 66 matched pairs of patients in this cohort, proned patients had lower mortality and longer duration of ECMO.

## Complications during prone positioning in ECMO patients

One of the main reasons for not proning patients who are receiving ECMO is the risk of ECMO-related complications, which could be fatal. The most dangerous complications are ECMO cannula dislodgment or a sudden decrease in blood flow. From the analyzed studies, four reported no relevant complications [20, 22, 25, 26]. Others reported minor complications [21, 23, 24], such as minor bleeding at the cannula insertion site and a temporary decrease in ECMO blood flow, which responded to fluid administration. Occasionally, endotracheal tube occlusion or ECMO membrane thrombosis has been reported. In the largest study analyzed, six prone position maneuvers needed to be aborted because of the appearance of respiratory or hemodynamic instability during the procedure [24].

One recent review that included 49 patients from seven different studies demonstrated that the development of complications during the proning of ECMO patients was very limited [27]. More importantly, all adverse events were rapidly and successfully reversed. In fact, they reported no cases of ECMO cannula dislodgment or chest tube or airway dislodgment.

## Which ECMO patients should be proned?

There are three possible answers to this question. The first is that ECMO patients should not be proned. Possible arguments to support this are the fact that they were proned prior to ECMO but no oxygenation improvement was observed, and the potential increased risk of complications during proning. Nevertheless, it should be noted that the benefits of the prone position beyond oxygenation improvements are well described and widely accepted. Moreover, when the maneuver is performed adequately, the incidence of complications during the treatment of ECMO patients has not been demonstrated to be higher than that in non-ECMO patients.

The second possible answer is that only a select group of patients should be proned. However, this implies that we need to define which ECMO patients would benefit the most from proning. In this sense, some authors decided to prone patients with dorsal infiltrates on computed tomography (CT) [20] as one may expect that they have a more heterogeneous ventilation distribution in the supine position and, therefore, would benefit most from proning. In fact, a greater improvement in compliance has been described in patients with a lower dorsal tidal volume/global tidal volume ratio [25]. Therefore, this approach emphasizes the change in the paradigmof prone position indication in patients with ARDS, moving from gas exchange criteria to lung mechanics criteria. In contrast, other studies that demonstrated the presence of approximately 50% of non-aerated or poorly aerated lung parenchyma on the CT scan of ECMO patients who were proned [18] found no correlation between CT scan findings and Crs and oxygenation after proning [18].

Finally, one could argue that all patients with ARDS who are receiving ECMO should be proned. This idea could be supported by the fact that the prone position has been shown to increase the survival of non-ECMO patients with ARDS [9].

**Fig. 1:**
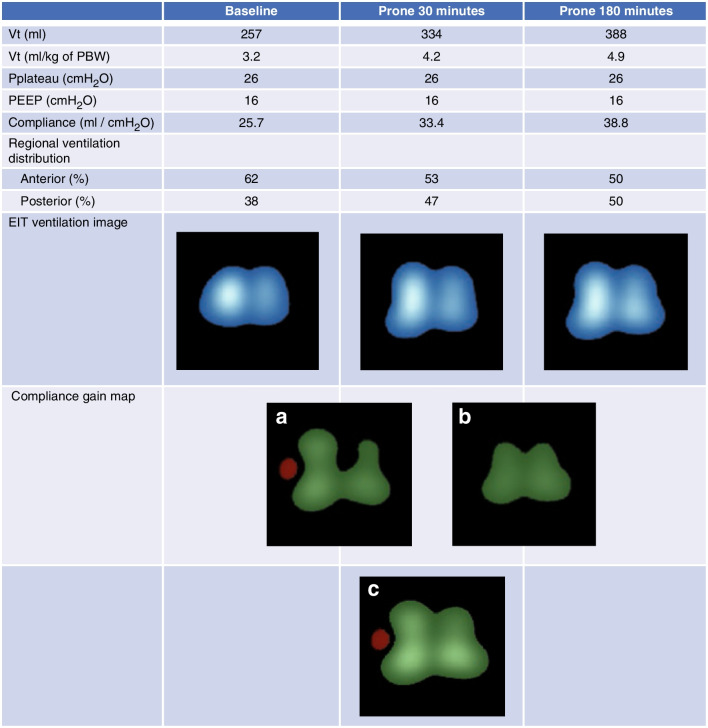
Example of the changes in pulmonary mechanics and regional ventilation distribution observed in one patient with acute respiratory distress syndrome (ARDS) treated with extracorporeal membrane oxygenation (ECMO) and ventilated with pressure-control ventilation. (**a**) represents the change in compliance observed between supine position and 30 min after prone position; (**b**) represents the variation in compliance between 30 and 180 min after prone position, and (**c**) represents the change in compliance between supine and 180 min after proning. *Green area* represents compliance gain and *red region* represents compliance loss. *Vt* tidal volume, *PBW* predicted body weight, *Pplateau* plateau pressure, *PEEP* positive end-expiratory pressure, *EIT* electrical impedance tomography

Second, it is worth noting that most of these patients had a preferred distribution of tidal ventilation to the ventral zones in the supine position; therefore, they could benefit from homogenizing lung inflation (Fig. 1). Moreover, as this increase in lung homogeneity was also present in patients with lower Crs and was independent of the mechanical response generated, it has been suggested that all ARDS patients who are receiving ECMO should benefit from proning [25].

Although it has been recently shown that the prone position may reduce inspiratory effort during spontaneous breathing in non-ECMO ARDS patients [16], the prone position is usually associated with the use of neuromuscular blockade and deeper sedation, avoiding spontaneous breathing. Conversely, the European Life Support Organization guidelines recommend an early reduction in sedation levels and a switch to spontaneous breathing after 24–48 h of ECMO initiation [28]. It is important to highlight that when this strategy is implemented one should be aware that monitoring respiratory drive and inspiratory effort [29] is strongly recommended to minimize the risk of patient self-inflicted lung injury. Indeed, it has been shown that around 50% of ARDS patients on ECMO present injurious inspiratoryeffort despite increasing sweep gas flows [30].

## Research priorities

Several questions remain unanswered, so there is a lot of room for improvement in this field. The evidence is mainly based on physiological or observational studies that included a small number of patients and studies in ARDS patients not receiving ECMO. Large randomized controlled trials (RCTs) are therefore needed to establish the role of the prone position in ARDS patients treated with ECMO. One of the most important unanswered questions is which ECMO patients would benefit from proning. It is also important to know about the relevance of timing of proning, as controversial results exist regarding the effectiveness of early and late proning [18, 26]. Finally, the duration of proning sessions is also important, as some data suggest that the benefits of the prone position may continuously increase beyond 16 h [18, 25].

Currently, two large RCTs have been designed to analyze the effect of the prone position on ARDS patients treated with ECMO. The first study (ClinicalTrials.gov Identifier NCT04139733) is designed to address the effect of early proning on the duration of ECMO. The second study (ClinicalTrials.gov Identifier NCT04607551) aims to analyze the effects of proning on weaning from ECMO.

## Conclusion

Use of the prone position has been shown to improve the survival of patients with moderate-to-severe ARDS. The results of observational studies have demonstrated that the prone position in ARDS patients treated with ECMO can be safely performed and has many physiological benefits that may potentially lead to a decrease in mortality. However, several questions remain unanswered and large RCTs that address the effectiveness of proning ECMO patients are still needed.

## Data Availability

Not applicable.
